# Regional Differences in Heat Shock Protein 25 Expression in Brain and Spinal Cord Astrocytes of Wild-Type and SOD1 ^G93A^ Mice

**DOI:** 10.3390/cells10051257

**Published:** 2021-05-19

**Authors:** Rebecca San Gil, Benjamin E. Clarke, Heath Ecroyd, Bernadett Kalmar, Linda Greensmith

**Affiliations:** 1 Molecular Horizons and School of Chemistry and Molecular Bioscience, Illawarra Health and Medical Research Institute, University of Wollongong, Wollongong, NSW 2519, Australia; r.sangil@uq.edu.au (R.S.G.); heathe@uow.edu.au (H.E.); 2 Neurodegeneration Pathobiology Laboratory, Queensland Brain Institute, University of Queensland, Brisbane, QLD 4072, Australia; 3 Department of Neuromuscular Disease, UCL Queen Square Institute of Neurology, London WC1N 3BG, UK; benjamin.clarke.15@ucl.ac.uk (B.E.C.); b.kalmar@ucl.ac.uk (B.K.); 4 The Francis Crick Institute, 1 Midland Road, London NW1 1AT, UK

**Keywords:** heat shock response, heat shock protein 27, glia, astrocytes, amyotrophic lateral sclerosis, motor neuron disease

## Abstract

Heterogeneity of glia in different CNS regions may contribute to the selective vulnerability of neuronal populations in neurodegenerative conditions such as amyotrophic lateral sclerosis (ALS). Here, we explored regional variations in the expression of heat shock protein 25 in glia under conditions of acute and chronic stress. Hsp27 (Hsp27; murine orthologue: Hsp25) fulfils a number of cytoprotective functions and may therefore be a possible therapeutic target in ALS. We identified a subpopulation of astrocytes in primary murine mixed glial cultures that expressed Hsp25. Under basal conditions, the proportion of Hsp25-positive astrocytes was twice as high in spinal cord cultures than in cortical cultures. To explore the physiological role of the elevated Hsp25 expression in spinal cord astrocytes, we exposed cortical and spinal cord glia to acute stress, using heat stress and pro-inflammatory stimuli. Surprisingly, we observed no stress-induced increase in Hsp25 expression in either cortical or spinal cord astrocytes. Similarly, exposure to endogenous stress, as modelled in glial cultures from SOD1 ^G93A^-ALS mice, did not increase Hsp25 expression above that observed in astrocytes from wild-type mice. In vivo, Hsp25 expression was greater under conditions of chronic stress present in the spinal cord of SOD1 ^G93A^ mice than in wild-type mice, although this increase in expression is likely to be due to the extensive gliosis that occurs in this model. Together, these results show that there are differences in the expression of Hsp25 in astrocytes in different regions of the central nervous system, but Hsp25 expression is not upregulated under acute or chronic stress conditions.

## 1. Introduction

In the healthy central nervous system (CNS), glia undertake several support functions that are vital for normal neuronal function and survival [1]. Increasing evidence suggests that both astrocytes and microglia exhibit molecular and functional regional heterogeneity, likely corresponding to the specific requirements of surrounding neuronal subtypes [2,3,4,5]. These regional differences in glia may also play an important role in the pathology of neurodegenerative diseases such as amyotrophic lateral sclerosis (ALS) [6]. ALS is a progressive neurodegenerative disorder affecting upper motor neurons in the primary motor cortex and lower motor neurons in the spinal cord, leading to their degeneration and subsequent muscle denervation and atrophy, resulting in premature death, typically within 2–5 years of diagnosis. Although ALS is primarily a disease of motor neurons, it is now well established that it is a non-cell-autonomous disease in which both astrocytes and microglia play an important role in the disease pathomechanism [7,8,9]. Both the loss of the supportive functions of glia [10,11] and gain of toxic immune phenotypes [12,13,14] are thought to contribute to motor neuron death in ALS. Existing regional differences in glia may further contribute to these mechanisms [11,15].

Since glia contribute to motor neuron death in ALS, it has been suggested that restoration of the lost supportive functions of glia or a reduction in the release of toxic factors from glia may be viable therapeutic strategies [16]. Targeting of heat shock proteins (Hsps), a family of cytoprotective protein chaperones, has been examined as a possible therapeutic approach in ALS, as some Hsps have both anti-aggregation and anti-inflammatory effects [15,17]. Indeed, amplification of the heat shock response (HSR) has shown promise in both pre-clinical and clinical ALS studies [18,19,20].

The small heat shock protein Hsp27 (*HSPB1*), with its murine homologue Hsp25, plays a crucial role in a multitude of cellular processes, including its primary role as a protein chaperone [21,22] as well as in regulation of cell growth, differentiation and cytoskeletal organization [23,24,25]. Anti-apoptotic, pro-survival properties of Hsp27 have also been widely reported in neurons as well as in tumor cells [26,27,28,29,30]. Furthermore, Hsp27 has been shown to reduce pro-inflammatory signalling through an interaction with I kappa B (Iκβ) kinase [31]. Elevated Hsp27 levels are observed under a wide range of stress conditions and are associated with increased neuronal survival and resistance to cell death [28,32,33,34,35]. Furthermore, aggregation of disease-linked proteins is prevented by elevated Hsp27 expression in vitro, in models which include Parkinson disease-linked alpha synuclein and ALS-linked mutant superoxide dismutase 1 (SOD1) [35,36,37].

In this study, we investigated the role of Hsp25 in mixed glial cultures obtained from different regions of the CNS in wild-type and SOD1 ^G93A^-ALS mice. Considering the diverse protective roles of Hsp27 in neuronal survival, we hypothesized that region-specific differences in Hsp27 expression might contribute to the differential vulnerability of motor neurons in SOD1 ^G93A^ mice. We also examined Hsp25 expression under basal conditions and following exposure to ALS-relevant cellular stress in primary murine mixed glial cultures from the spinal cord and cortex. Our results suggest that regional differences in the expression of Hsp25 expression exists between cortical and spinal cord astrocytes and that, surprisingly, Hsp25 expression is not altered in response to either acute or chronic stress.

## 2. Materials and Methods

### 2.1. Breeding and Maintenance of (C57BL/6 × SJL) F1 Hybrid WT and SOD1 ^G93A^ Mice

All mice were housed in a controlled temperature and humidity environment and maintained on a 12 h light/dark cycle with access to food and water provided ad libitum. All experiments were performed under license from the UK government in accordance with the Animals (Scientific Procedures) Act 1986 and following approval from the Institute of Neurology Ethical Review Committee. Non-transgenic, (C57BL/6 × SJL) F1 hybrids, and transgenic mice expressing human SOD1 ^G93A^ were obtained from Jackson Laboratories (USA). To model a chronic ALS related stress the SOD1 ^G93A^ mouse model was used. The SOD1 ^G93A^ mice (TgN [SOD1-G93A]1Gur) have been previously characterised to carry 25 copies of the SOD1 ^G93A^ transgene. The mice were maintained at the Institute of Neurology, University College London by breeding heterozygous male carriers with female (C57BL/6 × SJL) F1 hybrids.

### 2.2. Genotyping

The mice carrying the SOD1 ^G93A^ transgene were identified by polymerase chain reaction amplification of the human SOD1 transgene from genomic DNA. DNA was extracted from tail biopsies by incubating the biopsies at 55 °C/5 min in digest buffer, 10 mM Tris-HCl pH 8.3, 50 M KCl, 0.1 mg/mL gelatin, 0.45% (*v*/*v*) Nonidet P-40, 0.45% (*v*/*v*) Tween-20, supplemented with 1 U of proteinase K. Samples were then heated at 95 °C/10 min to inhibit proteinase K activity and centrifuged 23,000× *g*/2 min. The extracted genomic DNA in the supernatant was used to PCR amplify the human SOD1 transgene (forward primer: 5′-catcagccctaatccatctga-3′ and reverse primer: 5′-cgcgactaacaatcaaagtga-3′) and mouse interleukin-2 gene (forward primer: 5′-ctaggccacagaattgaaagatct-3′ and reverse primer: 5′- gtaggtggaaattctagcatcatc-3′) as an internal control.

### 2.3. Primary Murine Cortical and Spinal Cord Mixed Glial Cultures

Cortical and spinal cord primary mixed glial cultures were prepared from postnatal day 1–3 newborn (C57BL/6 × SJL) F1 mice using a protocol modified from McCarthy and de Vellis, 1980. Briefly, spinal cords and cortices were pooled separately in Ca^2+^/Mg^2+^ free Hank’s balanced salt solution (HBSS), cut into pieces with a scalpel, and mechanically dissociated by trituration. The tissue was enzymatically dissociated with 0.025% (*v*/*v*) trypsin, 0.02% (*w*/*v*) DNase I, 0.3% (*w*/*v*) BSA, 1% (*v*/*v*) penicillin/streptomycin in HBSS (37 °C/10 min) and proteolysis was inhibited with fetal calf serum (FCS). Cell suspensions were made by triturating 15 times and 5 mL FCS was added prior to centrifugation (400× *g*/5 min). The cell pellet was reconstituted with glial feeding medium, 15% (*v*/*v*) FCS, 1% (*v*/*v*) penicillin/streptomycin in DMEM and filtered through a 100 µm nylon mesh strainer. Cell suspensions were plated at a density of 2.5 × 10^5^ cells/mL into 6-well plates or glass coverslips in 24-well plates coated with poly-D-lysine. The glial feeding medium was changed 24 h post-plating and every 3 days thereafter and cultures were maintained at 37 °C under 5% CO_2_/95% air. After 12 days in vitro, the monolayer of cells reached 80–90% confluency and the composition of the resulting cortical and spinal cord mixed glial cultures were assessed by immunocytochemistry and flow cytometry by staining with anti-GFAP (astrocytic marker) and anti-CD11b or anti-Iba1 (microglial markers). Biological replicates of cultures for these studies were prepared from separate litters of newborn mice.

### 2.4. Treatment of Primary Mixed Glial Cultures

Primary mixed glial cultures were treated after 12 days in vitro with acute stressors: inflammatory mediators, LPS or TNFα, or heat shock (42 °C/30 min with recovery at 37 °C/6–24 h). Cells were treated for 24 h with 80 µg/mL LPS or 100 ng/mL TNFα diluted in glial feeding medium, concentrations that were shown to elicit a maximal inflammatory response in cortical and spinal cord primary mixed glial cultures. After treatment, cells were either harvested for immunoblot analysis or fixed in 4% (*w*/*v*) PFA for immunolabelling and subsequent epifluorescence microscopy or flow cytometry.

### 2.5. Protein Extraction and Quantification

Mixed glial cultures were washed twice with HBSS post-treatment. Whole-cell lysates were prepared by adding ice-cold RIPA buffer to each well [50 mM Tris (pH 7.5), 150 mM NaCl, 1% (*v*/*v*) NP-40, 0.5% (*w*/*v*) sodium deoxycholate, 0.1% (*w*/*v*) SDS, 1 mM EDTA, 1 mM EGTA, 1 mM PMSF, 1 × protease/phosphatase inhibitor cocktail] and harvesting the lysates on ice. The total protein (mg/mL) concentration of each sample was quantified by DC protein assay (Bio-Rad) according to the manufacturer’s instructions. Each sample was adjusted to 1 mg/mL with RIPA buffer to ensure equal protein loading of the SDS-PAGE for subsequent immunoblotting.

### 2.6. Antibodies

The antibody catalogue numbers and the concentrations used for immunoblotting (IB), immunocytochemistry (ICC) and flow cytometry (FC) are provided in parentheses. Goat polyclonal anti-Hsp27/Hsp25 (sc-1049; IB 1:1000, ICC 1:100, FC 1:100) was from Santa Cruz Biotechnology. Rabbit polyclonal anti-GFAP (ab7260; IB: 1:10,000, ICC: 1:1000) and β-actin (ab8226; IB: 1:40,000) was from Abcam. Mouse monoclonal anti-α-tubulin (A11126; IB: 1:1000) was from Thermo Fisher Scientific. Rabbit polyclonal anti-Iba1 (019-19741, IB 1:5000, ICC 1:500) was from Wako Laboratory Chemicals and rat polyclonal anti-CD11b-APC/Cy7 (101226, FC 1:1000) was from Biolegend. Mouse monoclonal anti-GFAP-Cy3 (C9205, ICC 1:500, FC 1:500) was from Sigma-Aldrich. Donkey anti-rabbit IgG-AlexaFluor488 (A21206, ICC 1:1000), anti-goat IgG-AlexaFluor488 (A11055, ICC 1:1000, FC 1:1000) secondary antibodies were from Thermo Fisher Scientific. Rabbit anti-mouse IgG-HRP (P0260, IB 1:1000), anti-goat IgG-HRP (P0160, IB 1:2000), and pig anti-rabbit-HRP (P0217, IB 1:2000) secondary antibodies were from Dako-Agilent Technologies.

### 2.7. Immunoblotting

Whole-cell lysates were resolved by SDS-PAGE using standard procedures. SDS protein extracts were electroblotted onto polyvinylidene difluoride membrane (PVDF; Bio-Rad) using a standard technique. Briefly, protein transfer to the PVDF membrane was performed in ice-cold immunoblotting transfer buffer (0.192 M glycine, 25 mM Tris, 20% (*v*/*v*) methanol, pH 8.6) at 100 V for 90 min. Membranes were blocked with 5% (*w*/*v*) non-fat milk in Tris-buffered saline (TBS; 50 mM Tris and 150 mM NaCl, pH 7.5) supplemented with 0.05% (*v*/*v*) Tween-20 (TBS-T) for 1 h at room temperature. Membranes were incubated at 4 °C overnight with 5% (*w*/*v*) non-fat dry milk in TBS-T and primary antibodies. The blots were washed four times for 10 min with TBS-T, incubated in 5% (*w*/*v*) non-fat dry milk in TBS-T with horseradish peroxidase -conjugated secondary antibody for 1 h at room temperature, and washed again in TBS-T four times for 10 min. Immunolabelled proteins were detected using Luminata Crescendo Western HRP substrate (Merk-Millipore) and chemiluminescence was captured using the Chemidoc Touch (Bio-Rad).

### 2.8. Immunolabelling

Cells were fixed in 4% (*w*/*v*) paraformaldehyde for 15 min either in suspension for flow cytometry or in a monolayer on a coverslip for epifluorescence and confocal microscopy. Cells were washed twice in PBS (pH 7.4) and then permeabilised and blocked in 5% (*v*/*v*) normal goat or donkey serum (or BSA) and 0.1% (*v*/*v*) TritonX-100 in PBS (PBS-T) for 1 h at room temperature. Cells were then incubated with primary antibodies diluted in 5% (*v*/*v*) normal goat or donkey serum or BSA in PBS-T for 16 h at 4 °C. The cells were washed three times in PBS for 5 min, incubated with secondary antibodies for 1 h at room temperature, and washed three times with PBS for 5 min. Cells destined for epifluorescence or confocal microscopy were counterstained with DAPI (1:1000 dilution) in PBS for 10 min at room temperature and washed three times in PBS for 5 min. Coverslips were mounted onto 26 × 76 mm glass slides (Thermo Fisher Scientific) using Citifluor™ Anti-Fadent Mounting Solutions (ProSciTech) for epifluorescence or confocal microscopy.

### 2.9. Epifluorescence Microscopy

Mixed glial cortical and spinal cord cultures were plated on 10 mm coverslips, fixed and immunolabelled post-treatment. The slides were analysed using a Leica epifluorescence microscope system with Leica Application Suite v.2.8.1 software, using the 20× or 40× objective lens. AlexaFluor488 fluorescence was excited using a mercury lamp with a 488 nm filter and Cy3 fluorescence was excited using a 561 nm filter. Images were captured using a Nikon colour camera. The proportion of cells that were Hsp25-positive were determined using MetaMorph Image Analysis v7 software (Molecular Devices, San Jose, CA, USA) and Equation (1).

$$\left(\frac{\#\;Hsp25\;positive\;of\;GFAP\;positive}{Total\;\#\;GFAP\;positive}\right)\times 100=\%Hsp25\;positive\;astroglia$$

### 2.10. Confocal Microscopy

The slides were analysed using a Zeiss 510 confocal microscope using the 63× oil-immersion objective lens (Zeiss, Oberkochen, Germany). Fluorescence was excited at 405, 488, and 561 nm to image DAPI, AlexaFluor488, and Cy3, respectively. Fluorescent emissions from fluorophore-conjugated secondary antibodies were acquired by sequential scanning using the Zen 2013 software.

### 2.11. Flow Cytometry

After fixing and immunolabelling, cells were resuspended in 200 µL of PBS for single-cell analysis by flow cytometry. Each set of samples (untreated, inflammatory mediator treated, and heat shocked samples) were analysed together across three biological replicates. Flow cytometry was performed using a FACS Aria II (BD Biosciences, San Jose, CA, USA). A minimum of 20,000 events per sample were collected. Forward scatter and side scatter were collected using a linear scale, and fluorescent emissions were collected as log scale for each channel. For AlexaFluor488 fluorescence, data were collected with the 488 nm laser and 530/30 bandpass filter. For Cy3 fluorescence, data were collected with the 561 nm laser and 582/15 bandpass filter. For APC/Cy7 fluorescence, data were collected with the 633 nm laser and the 670 longpass filter. 

### 2.12. Statistics

Results shown are the mean ± S.E.M. of three independent experiments (three litters of newborn mice). Evaluation of differences in means was determined by Student’s *t*-test, a one-way analysis of variance (ANOVA) or two-way ANOVA, where indicated. The F-statistic from the ANOVA test, associated degrees of freedom (between groups and within groups, respectively), and the *p*-value from the ANOVA test are shown in parentheses. Post hoc testing of differences between means was done using Bonferroni’s test using GraphPad Prism 5 (GraphPad Software, Inc., La Jolla, CA, USA).

## 3. Results

### 3.1. Characterisation of Cortical and Spinal Cord Mixed Glial Cultures

Primary glial cultures were derived from the cerebral cortex and spinal cords of postnatal day 2–3 mice. To determine whether cortical and spinal cord mixed glial cultures were composed of comparable proportions of astrocytes and microglia, cultures from both regions were analysed by immunoblot and immunofluorescence (Supplementary Figure S1). Immunoblot analysis of the cortical and spinal cord mixed glial cultures revealed that in both cultures, there was a relatively higher expression of the astrocytic marker GFAP compared to the microglial marker Iba1 (Supplementary Figure S1a,b). This higher level of expression of astrocytes compared to microglia in cortical and spinal cord glial cultures was confirmed by GFAP and Iba1 immunolabelling and subsequent fluorescence microscopy (Supplementary Figure S1c).

### 3.2. Expression of Hsp25 in Cortical and Spinal Cord Mixed Glial Cultures

Primary cortical and spinal cord glial cultures were treated with ALS-relevant inflammatory stimuli to assess their capacity to induce Hsp25 expression after acute stress. It has previously been shown that treatment of glial cultures with pro-inflammatory stimuli such as lipopolysaccharide (LPS), results in the induction of the HSR, as determined by an increase in Hsp27 expression [38]. In order to examine whether the differential expression of Hsp25 in different brain regions had implications for the stress response of astrocytes in the spinal cord and cortex, we examined Hsp25 expression following treatment of mixed glial cultures with the inflammatory mediators LPS, a bacterial endotoxin that activates the NF-kB-mediated inflammatory pathway [39], or tumor necrosis factor a (TNFα), a pro-inflammatory cytokine previously shown to be upregulated in response to mutant SOD1 [40,41,42,43].

**Figure 1:**
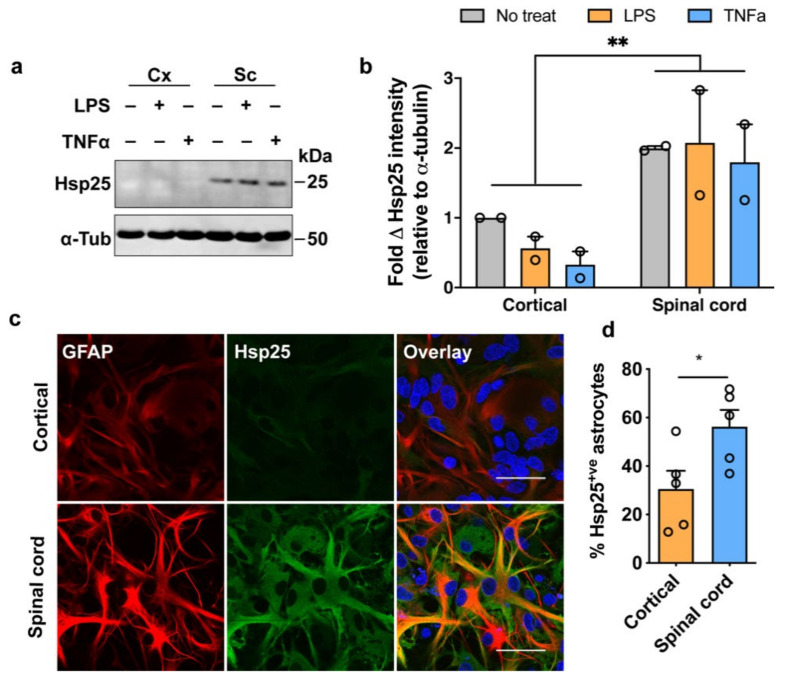
Hsp25 expression in LPS- or TNFα-stimulated primary cortical and spinal cord glial cultures. (**a**) Mixed primary cortical (Cx) and spinal cord (Sc) glia were treated with 80 µg/mL LPS or 100 ng/mL TNFα for 24 h. 30 µg of total protein was immunoblotted and probed for Hsp25 (25kDa) and α-tubulin (50 kDa). (**b**) Fold change in Hsp25 band intensity relative to the untreated cortical sample and normalised to α-tubulin to account for differences in protein loading. Data presented are the mean ± SEM from three biological replicates. Statistically significant differences between the means were assessed using a two-way ANOVA followed by Bonferroni’s post hoc test. (**c**) Representative images of untreated cortical and spinal cord glial cultures immunolabelled for GFAP (astrocytic marker; left) and Hsp25 (middle). Overlay (right) is shown with DAPI nuclear stain. Scale bar = 30 µm. (**d**) The proportion of Hsp25^+ve^ astrocytes from cell counts of cortical and spinal cord glial cultures. Data shown are from one biological replicate and are the mean ± SEM of >300 cells across five wide field of view images. Statistically significant differences between the means were assessed using Student’s *t*-test, where *p* < 0.05 (*), *p* < 0.01 (**).

Immunoblot analysis of Hsp25 showed significantly higher levels of the Hsp25 protein in spinal cord glial cultures compared to cortical cultures, even in the absence of any stressful stimuli, with Hsp25 expression in the spinal cord 1.9 times greater than that in cortical cultures [F (1, 6) = 17.07, *p* = 0.006] (Figure 1a,b). However, there was no stress-induced upregulation in Hsp25 in response to either LPS or TNFα treatment in either cortical or spinal cord cultures (Figure 1a,b). Further investigation of Hsp25 expression in cortical and spinal cord mixed glial cultures by immunocytochemistry supported the findings from the immunoblot analysis, with a higher level of Hsp25 immunoreactivity observed in spinal cord compared to cortical glial cultures, particularly in GFAP-positive astrocytes (Figure 1c). Cell counts of the proportion of Hsp25^+ve^ and GFAP^+ve^ astrocytes in each culture revealed that under basal conditions, 30 ± 7% of cortical astrocytes and 56 ± 6% of spinal cord astrocytes were Hsp25^+ve^ (Figure 1d; *p* = 0.0361). These findings show that there are regional differences in the basal level of Hsp25 expression, with a two-fold greater number of Hsp25^+ve^ astrocytes present in spinal cord compared to cortical cultures, although exposure to inflammatory stressors fails to activate a stress response in astrocytes from either region.

To further investigate regional differences in Hsp25 expression between cortical and spinal cord glia, the cultures were immunostained for the astrocytic marker GFAP, the microglia marker, CD11b, and Hsp25 for single-cell analysis by flow cytometry. Viable cells were gated based on their forward and side scatter to exclude cellular debris and cell clumps (Supplementary Figure S2a,b). With respect to analysing the GFAP^+ve^ cells, bisected polygonal gates were set using the unlabelled sample as a GFAP^-ve^ and Hsp25^-ve^ control, and two controls that were singly labelled for either GFAP alone or Hsp25 alone (Supplementary Figure S2d–f). Gating the flow cytometry data in this way allowed the proportion of Hsp25^+ve^ cells in the GFAP^+ve^ astrocyte population to be determined in both the cortical and spinal cord mixed glial cultures. Similarly, cells were either unlabelled or immunolabelled for CD11b or Hsp25 alone as single-colour controls (Supplementary Figure S2g–i). This gating strategy allowed the proportion of Hsp25^+ve^ cells of the CD11b^+ve^ microglial population to be determined in cortical and spinal cord mixed glial cultures.

**Figure 2:**
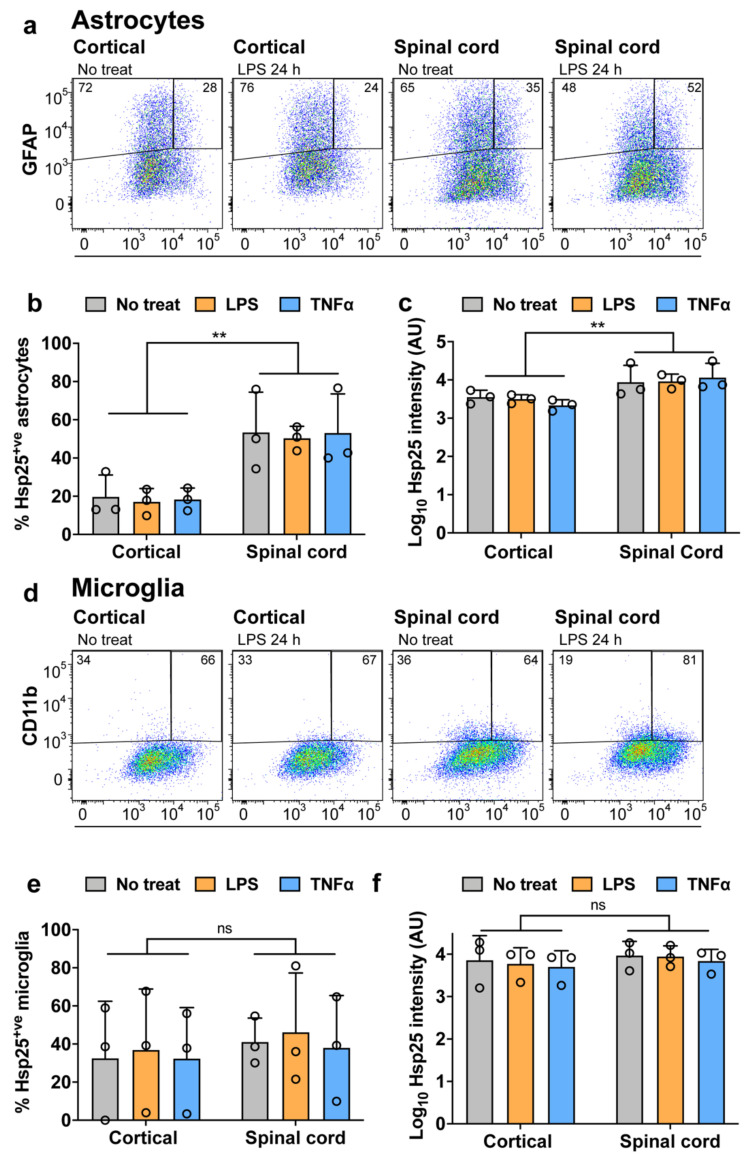
Flow cytometric analysis of Hsp25 expression in astrocytes and microglia after LPS and TNFα treatment. Primary cortical and spinal cord mixed glial cultures were treated with 80 µg/mL LPS or 100 ng/mL TNFα. The cells were fixed, permeabilised and immunolabelled for GFAP-Cy3, CD11b-APC/Cy7 and Hsp25-AF488. (**a**,**d**) Flow cytometric analysis of cortical and spinal cord glial cultures. Left–right: Representative cytograms of untreated and LPS treated cortical glial cultures, and untreated and LPS treated spinal cord cultures. Data are presented as pseudo-colour scatter plots where blue represents low and red represents a high frequency of cells. The percent of GFAP^+ve^ or CD11b^+ve^ cells in each gate is provided. (**b**,**e**) The percent of Hsp25^+ve^ astrocytes or microglia as determined from the flow cytometric analysis. (**c**,**f**) The Hsp25-AF488 fluorescent median of the astrocytic or microglial population in arbitrary units (AU). Data shown are the means ± SEM of three biological replicates. Differences between the means were determined using a two-way ANOVA followed by Bonferroni’s post hoc test, where *p* < 0.01 (**).

Representative cytograms of GFAP and Hsp25 double-immunolabelled cortical and spinal cord mixed glial cultures under basal conditions or following treatment with LPS are shown in Figure 2a. A two-way ANOVA confirmed that there was a significant effect of the CNS region of the cultures on the proportion of Hsp25-positive astrocytes [F (1, 18) = 18.08, *p* = 0.0005], with an approximately 2-fold increase in the proportion of spinal cord astrocytes expressing Hsp25 compared to cortical astrocytes (Figure 2b). This is in agreement with the increase in Hsp25 levels observed in the immunoblot analysis (Figure 1a,b). Treatment with either LPS or TNFα had no effect on either the proportion of cells expressing Hsp25 [F (2, 18) = 0.018, *p* = 0.9817] or the level of Hsp25 expressed in Hsp25-positive cells (Figure 2c).

Since many stress responses, including inflammatory reactions, are primarily attributed to microglia, we next examined the expression of Hsp25 in microglia, which are also present in the mixed primary cortical and spinal cord cultures. Representative cytograms of CD11b and Hsp25 immunolabelled spinal cord and cortical samples in the presence and absence of LPS treatment are shown in Figure 2d and were used to determine the proportion of Hsp25-positive microglia and the levels of Hsp25 expression in each of the samples (Figure 2e,f). Approximately 40% of CD11b^+ve^ spinal cord and cortical microglia were positive for Hsp25 across all samples and regardless of treatment. Under basal conditions, no differences were observed in either the proportion of Hsp25-positive microglia or the level of Hsp25 expression between spinal cord or cortical microglia. Treatment with LPS or TNFα had no effect on Hsp25 expression in either spinal cord or cortical microglia.

These results show that Hsp25 expression is higher in spinal cord cultures than in cortical cultures, primarily as a result of a 2-fold increase in the proportion of astrocytes expressing Hsp25 in spinal cord compared to cortical cultures. In contrast, there was no difference in the level of Hsp25 expression in spinal cord and cortical microglia. Furthermore, these findings also show that inflammatory stimuli do not upregulate the expression of Hsp25 in microglia or astrocytes, irrespective of regional location within the CNS.

**Figure 3:**
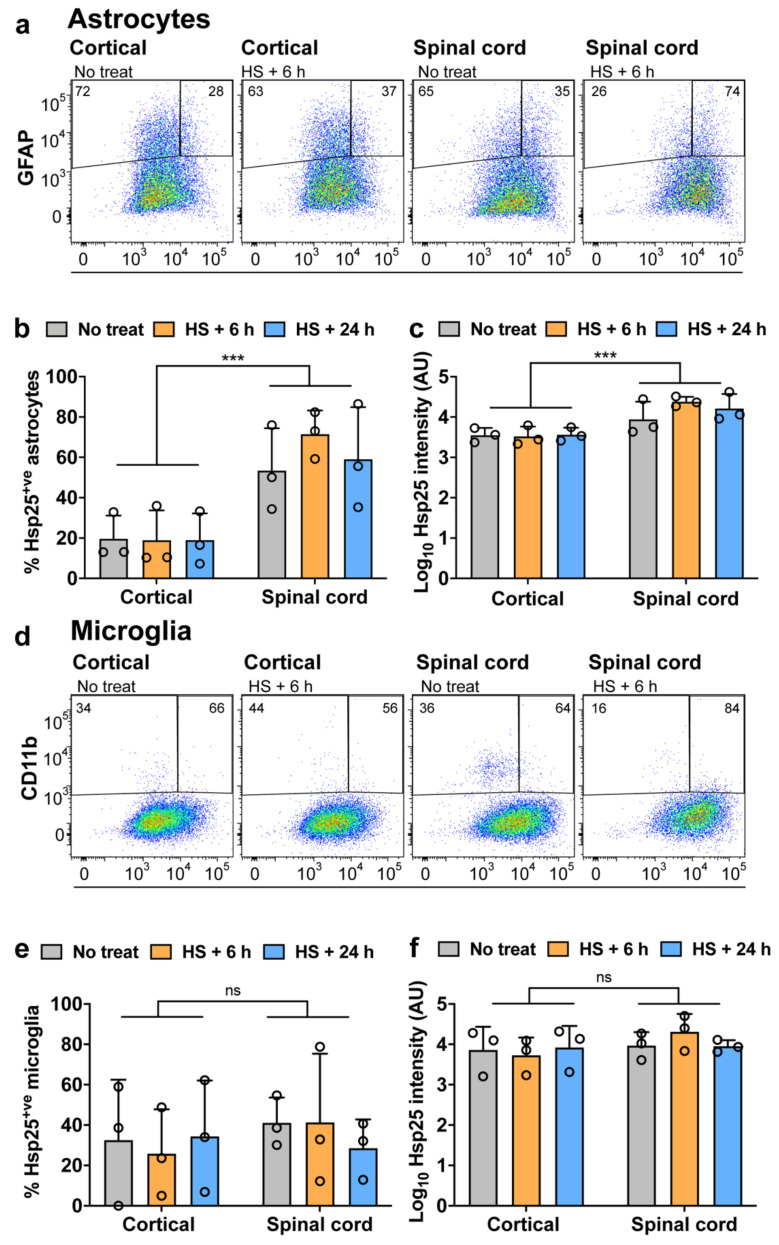
Flow cytometric analysis of Hsp25 expression in astrocytes and microglia after heat shock. Mixed primary cortical and spinal cord glia were heat shocked at 42 °C for 30 min and allowed to recover at 37 °C for 6 or 24 h. Cells were then fixed, permeabilised, immunolabelled for GFAP-Cy3, CD11b-APC/Cy7 and Hsp25-AF488 and analysed by flow cytometry. (**a**,**d**) Flow cytometric analysis of cortical and spinal cord glial cultures. Left–right: Representative cytograms of untreated and heat shocked cortical glial cultures, and untreated and heat shocked spinal cord cultures. Data are presented as pseudo-colour plots where blue represents low and red represents a high frequency of cells. Outlier events are shown as individual black dots. The percent of GFAP^+ve^ or CD11b^+ve^ cells in each gate is provided. (**b**,**e**) The percent of Hsp25^+ve^ astrocytes or microglia as determined from the flow cytometric analysis. (**c**,**f**) The median of the Hsp25-AF488 fluorescence intensity of the astrocytic or microglial population. Data shown are the means ± SEM of three biological replicates. Potential differences between the means were assessed using a two-way ANOVA followed by Bonferroni’s post hoc test, where *p* < 0.001 (***).

Since neither astrocytes nor microglia upregulated Hsp25 following treatment with either of the inflammatory mediators, the cultures were next subjected to heat shock (42 °C for 30 min followed by 6 or 24 h recovery at 37 °C) (Figure 3), which resulted in an upregulation of Hsp70 (Supplementary Figure S3). Surprisingly, heat shock had no effect on the proportion of astrocytes expressing Hsp25 in either spinal cord or cortical cultures, at either the 6 or 24 h recovery periods examined (Figure 3a–c). As can be seen in Figure 3b,c, there is a heat shock-induced increase in the proportion of Hsp25-positive spinal cord astrocytes at the 6 h recovery period, from 43 ± 17% in non-stressed cultures to 72 ± 7% following heat shock (Figure 3b,c), but this was not statistically significant [F (2, 12) = 0.6076, *p* = 0.5606; Figure 3c]. Analysis of microglia revealed a similar lack of response to heat shock, which had no effect on the proportion of Hsp25-positive microglia or Hsp25 expression levels in either the spinal cord or cortical samples (Figure 3d–f). These results therefore show that under basal conditions, a higher proportion of spinal cord astrocytes are Hsp25-positive compared to cortical astrocytes. Surprisingly, exposure to heat shock has no effect on the proportion of astrocytes or microglia that are Hsp25^+ve^, in either cortical or spinal cord samples. Taken together, with the findings of the effects of inflammatory mediators (Figure 2), these results suggest that irrespective of location within the CNS, neither astrocytes or microglia upregulate Hsp25 expression in response to acute stressful stimuli in vitro. Similar findings have been reported in human foetal astrocytes which also fail to upregulate Hsp27 in response to heat shock [44].

### 3.3. Hsp25 Expression in Spinal Cord and Cortical Glial Cultures Expressing SOD1 ^G93A^

**Figure 4:**
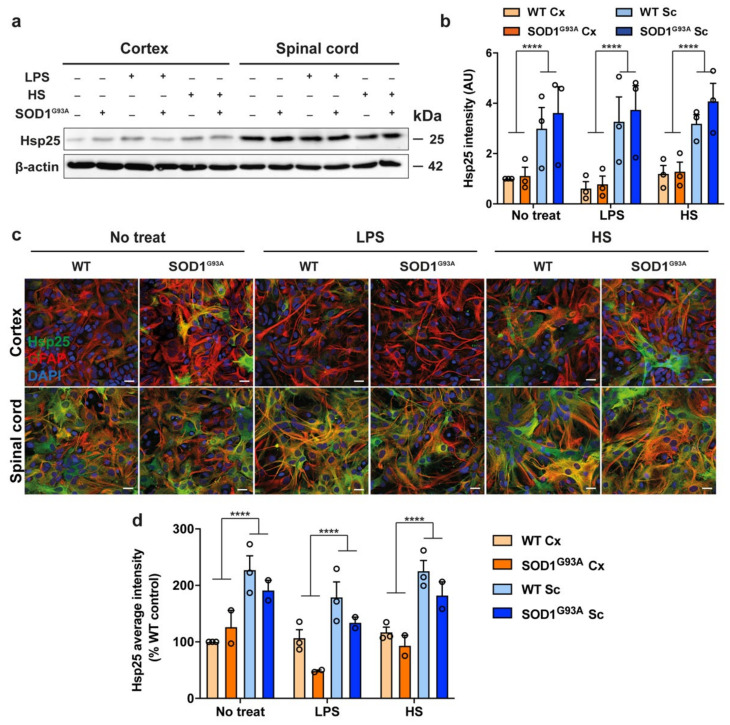
Hsp25 expression in SOD1 ^G93A^ mixed glial cultures derived from the cortex and spinal cord. Mixed primary cortical and spinal cord glia were either treated with LPS (80 µg/mL) or heat shocked (42 °C/30 min followed by recovery at 37 °C/24 h). Cells were then harvested for immunoblotting or fixed, permeabilised and immunolabelled for GFAP and Hsp25 and analysed by fluorescence microscopy. (**a**,**b**) Immunoblotting, quantification and fold change of Hsp25 (25 kDa) levels in whole-cell lysates relative to β-actin in cortical and spinal cord samples. (**c**) Immunofluorescence of mixed glial cultures immunolabelled for Hsp25 and GFAP and counterstained with DAPI. Scale bar = 20 µm. (**d**) The average intensity of Hsp25-AF488 staining normalised to WT cortical control in cortical and spinal cord samples. Data shown are the means ± SEM of 2–3 independent experiments. Statistically significant differences between the means were assessed using a two-way ANOVA followed by Bonferroni’s post hoc test, where *p* < 0.0001 (****).

Although acute exposure to inflammatory stressors failed to induce an upregulation in Hsp25 expression in either spinal cord or cortical astrocytes, or microglia, we next examined Hsp25 expression levels in spinal cord and cortical cultures derived from the well-characterised SOD1 ^G93A^ mouse model of ALS. To determine whether the regional difference in astrocytic Hsp25 expression observed under non-stress conditions (Figure 1) is altered in an ALS model, primary mixed SOD1 ^G93A^ spinal cord and cortical cultures were analysed by immunoblot and immunocytochemistry. Immunoblot analysis demonstrated a significant 2–4-fold increase in Hsp25 levels in spinal cord glia compared to cortical glia in both wild-type (WT) and SOD1 ^G93A^ cultures [F (3, 24) = 15.86, *p* < 0.0001; Figure 4a,b]. However, SOD1 ^G93A^ expression had no effect on the level of Hsp25 in either spinal cord or cortical cultures when compared to WT mixed glial cultures (Figure 4a,b). Exposure to additional acute stress by treatment of the cultures with LPS or heat shock had no effect on Hsp25 levels (Figure 4a,b). Within the brain region, there were no statistically significant differences between the Hsp25 intensity in WT and SOD1 ^G93A^ mixed glial cultures in any of the treatment conditions (Figure 4b). Hsp25 was expressed in GFAP^+ve^ astrocytes, as opposed to other cell types present in SOD1 ^G93A^ mixed glial cultures (Figure 4c). In addition, quantification of Hsp25 intensity levels demonstrated similar levels of Hsp25 expression in WT and SOD1 ^G93A^ spinal cord astrocytes, and this was approximately 2-fold greater than the expression levels in cortical astrocytes [F (3, 18) = 26.73, *p* < 0.0001; Figure 4d]. Within the brain region, there were no statistically significant differences between the Hsp25 intensity in WT and SOD1 ^G93A^ mixed glial cultures in any of the treatment conditions (Figure 4d). These findings suggest that the expression of the SOD1 ^G93A^ transgene combined with exposure to acute stressors do not alter the Hsp25 expression levels in spinal cord or cortical astrocytes. However, basal regional differences in the expression of Hsp25 were still observed, with spinal cord astrocytes expressing Hsp25 to a greater extent than cortical astrocytes.

### 3.4. Expression of Hsp25 in the Spinal Cord and Brain of Adult Wild-Type and SOD1 ^G93A^ Mice

**Figure 5:**
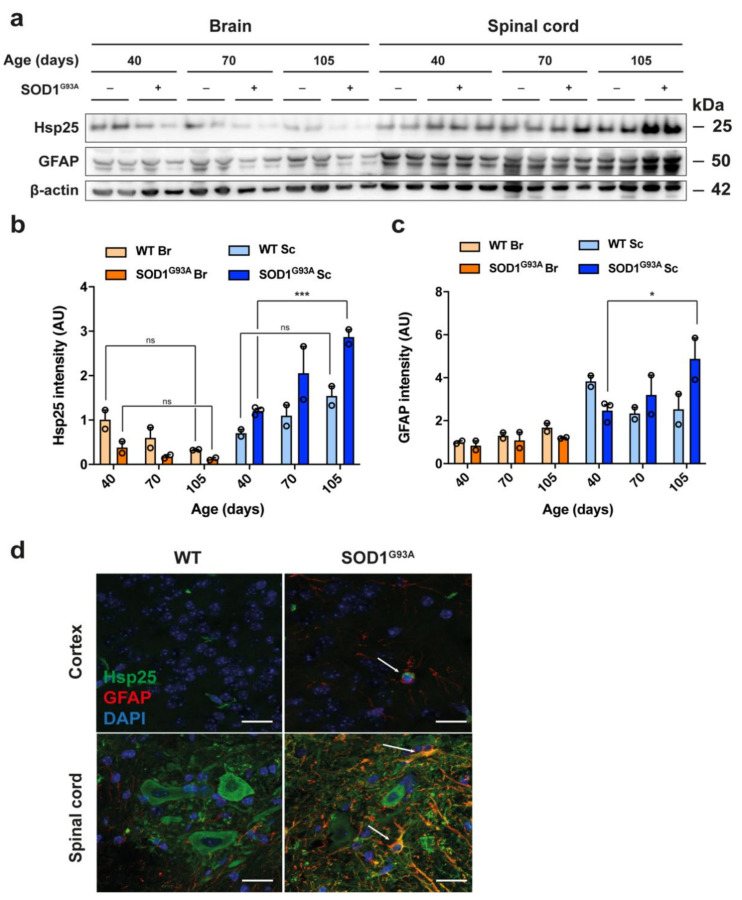
Hsp25 expression in the adult spinal cord and cortex of WT and SOD1 ^G93A^ mice. (**a**) Immunoblot analysis of Hsp25 and GFAP expression levels in whole brain and whole spinal cord lysates. (**b**) Quantification of Hsp25 and (**c**) GFAP expression in the brain and spinal cord of 40-, 75- and 105-day-old WT and SOD1 ^G93A^ mice. Data are displayed as the mean ± SEM of 2-3 biological replicates and analysed by two-way ANOVA with post hoc Tukey tests, where *p* < 0.05 (*) and *p* < 0.001 (***). (**d**) Immunofluorescence of layer V of the cortex and ventral horn spinal cord sections from 90 day old WT and SOD1 ^G93A^ mice. Scale bar: 20µm.

Since the process of culturing can in itself be a stressful stimulus to primary cells, it is possible that the level of Hsp25 expression detected within ‘unstressed’, untreated samples (Figure 1, Figure 2, Figure 3 and Figure 4) already reflects the cellular response to stress associated with culturing cells from primary tissue, such that exposure to additional stressors such as treatment with inflammatory mediators or heat shock or expression of SOD1 ^G93A^ may fail to increase Hsp25 levels further. We next examined Hsp25 expression in spinal cord and brain tissue from WT and SOD1 ^G93A^ mice. To determine the Hsp25 expression pattern in vivo under stress and non-stress conditions, whole brain and spinal cord lysates from WT mice as well as SOD1 ^G93A^ mice at a range of disease stages mice were examined by immunoblot, at 40 days (pre-symptomatic), 70 days (early symptomatic) and 105 days of age (late stage symptomatic). In 40-day-old adult mice, Hsp25 levels were ~1.5-fold greater in the spinal cord compared to the brain in WT and pre-symptomatic SOD1 ^G93A^ mice [F (5, 13) = 23, *p* < 0.0001; Figure 5a,b]. There was trend towards Hsp25 decline between 40–105 days in cortical samples from both WT (*p* = 0.69) and SOD1 ^G93A^ (*p* > 0.99) mice, but this was not significant (Figure 5a,b). The pattern of Hsp25 expression in the spinal cord was different from that observed in cortical samples, with a slight, albeit non-significant increase in Hsp25 expression in WT spinal cord samples between 40 and 105 days of age (*p* = 0.2696; Figure 5a,b), and a significant increase in Hsp25 levels in SOD1^G93A^ spinal cord from 40 to 105 days (*p* = 0.0008; Figure 5a,b). There was a significant increase in Hsp25 levels between WT and SOD1 ^G93A^ brain tissue at 70 days, an early symptomatic time point (*p* = 0.0072) and a trend towards greater Hsp25 levels at 105 days but this was not significant (*p* = 0.8708) (Figure 5b). This increase in Hsp25 expression in spinal cord samples of SOD1 ^G93A^ mice correlated with a significant increase in GFAP levels (*p* = 0.0251; Figure 5c), reflecting the well-characterised astrogliosis that is known to occur in the SOD1 ^G93A^ model of ALS [45,46,47]. There was a significant increase in GFAP levels between WT and SOD1 ^G93A^ brain tissue at 40 days, a pre-symptomatic timepoint (*p* = 0.0196) and 105 days, late stage symptomatic (*p* = 0.0035) (Figure 5c). These results suggest that although an upregulation in Hsp25 expression occurs in the spinal cord during disease progression in SOD1 ^G93A^ mice, this is likely to be due to the increase in GFAP-positive astroglia, since a significant astrogliosis is known to occur in the spinal cord of SOD1 ^G93A^ mice.

Immunostaining of 90-day-old symptomatic SOD1 ^G93A^ and age-matched WT mice showed that Hsp25 immunoreactivity was greater in ventral horn spinal cord sections than in layer V cortical sections (Figure 5d). Staining for GFAP, a marker of reactive astrocytes, revealed that there were no reactive astrocytic cell bodies in the cortex or ventral horn of WT mice. In contrast, in symptomatic 90-day-old SOD1 ^G93A^ mice there was an increase in GFAP labelling, indicating the presence of astrogliosis (Figure 5d). The GFAP-positive cells in the SOD1 ^G93A^ spinal cord were immunoreactive for Hsp25, as were the large cell bodies of motor neurons. These results show that in vivo, in both WT and SOD1 ^G93A^ mice, a greater proportion of cells in the spinal cord are Hsp25-positive than in the cortex, and these are largely comprised of astrocytes.

In summary, we confirm that both Hsp25 and GFAP are expressed to a greater extent in spinal cord than brain of both WT and SOD1 ^G93A^ mice, as observed in primary tissue harvested from these regions and examined in mixed glial cultures. In the cortex, Hsp25 expression remains largely stable with age in both WT and SOD1 ^G93A^ mice, in contrast to the spinal cord, in which there is a significant increase in Hsp25 expression in late stage symptomatic SOD1 ^G93A^ mice. There is no significant change in GFAP expression in the cortex of either WT or SOD1 ^G93A^ mice or in WT spinal cord, but there is a clear increase in GFAP levels in the spinal cord of SOD1 ^G93A^ mice. These findings confirm that Hsp25 expression increases during disease progression in SOD1 ^G93A^ mice, in cells under conditions of SOD1 ^G93A^ -induced stress, most likely as a result of the astrogliosis that is a characteristic feature of spinal cord pathology in the SOD1 ^G93A^ mouse model of ALS.

## 4. Discussion

ALS is a complex non-cell-autonomous disease, with spinal cord motor neurons, astrocytes and microglia each contributing to the development and progression of the disease. Although both upper and lower motor neurons are affected in ALS, the most prevalent pathology has been described in the spinal cord, with widespread loss of motor neurons accompanied by significant activation of astrocytes and microglia [48,49]. Increasing evidence shows that astrocytic and microglial transcriptomes, proteomes and subsequent functions differ across regions of the CNS and thus it is likely that glial support for neuronal function also differs in different CNS regions [5]. Differential functional characteristics may underlie the specific vulnerability of neuronal populations in neurodegenerative conditions such as that observed in ALS. We have previously shown that spinal cord glia have an exacerbated inflammatory response to pro-inflammatory stimuli, which might contribute to the development of harmful inflammatory environment in the spinal cord in ALS [15].

In ALS as well as in other neurodegenerative diseases, a prominent feature of the disease is the dysfunction of the proteostasis machinery, resulting in the appearance of characteristic intracellular protein aggregates [50,51,52]. Heat shock proteins are a large and diverse family of molecular chaperones, which play a role in transporting and folding nascent polypeptides into their native conformation and detecting, refolding or degrading misfolded proteins. It has been shown that the cellular availability of several classes of Hsps progressively decreases with age and this creates a vulnerability in long lived post mitotic cells like neurons [53]. Indeed, we have found that under heat stress conditions there is a significant downregulation of Hsp70 in primary glial cells obtained from mice harbouring the ALS causing SOD1 ^G93A^ mutation [15]. The family of small Hsps, which includes Hsp25, are ATP-independent but are still able to stabilise misfolded proteins and reduce aggregation of disease-causing protein species [35,36,37]. Hsp25 is a constitutively expressed molecular chaperone in motor neurons and plays important roles in “housekeeping” and stress-induced proteostasis in the CNS. In view of our previous findings regarding region-specific inflammatory and Hsp70 responses in spinal cord and cortical glia, in this study, we investigated the regional patterns of expression and stress-inducible upregulation of Hsp25.

The results presented in this study show (i) that subsets of the astrocyte population in the cortex and spinal cord are Hsp25-positive under basal conditions and (ii) that there are double the proportion of Hsp25-positive astrocytes in primary spinal cord glia compared to cortical glial cultures; these findings align with previous work in human post-mortem tissue [54]. Importantly, we also show that neither cortical nor spinal cord mixed glial cultures up-regulate the expression of Hsp25 after treatment with inflammatory mediators, indicating that disease-associated inflammatory insults may not be sufficient to upregulate the expression of Hsp25 in astrocytes and microglia. Using the SOD1 ^G93A^ mouse model of ALS, we also show that the expression of mutant SOD1 does not affect the regional pattern of Hsp25 expression in primary glial cultures. This finding indicates that there are differences between stress responses of Hsp70 and Hsp25 expression using the same experimental paradigms, indicating that the HSR is composed of various elements contributing to tissue- and stress-specific responses. In the present study, we found that the overall expression of Hsp25 in SOD1 ^G93A^ mice is elevated at late-stage disease. However, this marked increase in Hsp25 expression is limited to the spinal cord and is absent in brain tissues of the same transgenic mice, indicating that the spinal cord environment may be more prone to the damage caused by ALS-causing mutations, such as SOD1 ^G93A^, than the cortex. This increase in Hsp25 expression is likely due to the astrogliosis that occurs as disease progresses in this mouse model. Further investigation of the role of Hsp25 in astrogliosis and in disease progression in the SOD1 ^G93A^ mouse would be of interest to study in a future study.

This study also showed that exposure of mixed glial cultures to inflammatory mediators does not upregulate Hsp25 expression. Immunoblot and flow cytometric analysis showed that there was no change in Hsp25 levels following treatment with LPS or TNFα in either spinal cord or cortical astrocytes derived from either WT or SOD1 ^G93A^ mice. In contrast, treatment of primary rat mixed glial cells with increasing doses of a combination of LPS and IFNγ, has been reported to result in a concentration-dependent increase in both iNOS and Hsp70 [55]. The difference in the stress-induced expression of Hsp70 observed in the Calabrese study and the absence of an effect on Hsp25 expression in the present study suggests that astrocytes only induce some elements of the HSR in response to specific activation by certain inflammatory mediators or perhaps species dependent responses occur in these cells. For example, Hsp25 mRNA levels are increased in human cortical astrocytes after treatment with a mixture of inflammatory mediators comprised of IL-1β, IFNγ and TNFα [56]. However, Hsp25 mRNA levels did not change when treated with each of these inflammatory mediators individually, suggesting a synergistic mode of action of pro-inflammatory cytokines on Hsp25 transcription in these cells. In the present study, LPS and TNFα treatment was used to mimic the inflammatory stress that is present in the CNS of ALS patients, but was not sufficient to induce Hsp25 expression in astrocytes in any of the cultures tested in our experimental conditions.

Exposure to heat shock also failed to induce an upregulation in Hsp25 expression in either spinal cord or cortical glial cultures. However, we found that twice as many spinal cord astrocytes were Hsp25-positive than in cortical glial cultures, even in basal conditions in the absence of any stimuli. Previous studies have reported evidence of regional differences in stress-induced expression of Hsp25. Investigation of Hsp25 expression in rat astrocytes across various regions of the CNS have shown that Hsp25-positive astrocyte sub-populations are found in some hippocampal regions of rats with hyperthermia including CA1, CA3 and the dentate gyrus but not CA1, CA2 or the subiculum [57,58]. In another study, excitotoxic lesions resulted in an increase in Hsp25 in cortical astrocytes at 3–5 days, and in the thalamus at 5–7 days, suggesting differential regulation of Hsp25 in these two brain regions [59]. In the present study, we consistently found that a significant number of astrocytes in culture remained Hsp25-positive even though the level of GFAP expression in these cells was similar to that in non-Hsp25-positive cells. This indicates that Hsp25 may be used as a possible marker of a subclass of astrocytes that are enriched in the spinal cord. The practical implications of these findings suggest that the CNS region from which glia are derived should be carefully considered in studies that investigate interactions between spinal cord motor neurons and glia.

In conclusion, the results of this study demonstrate that there are significant differences between glia derived from the spinal cord and cortex with respect to Hsp25 expression. We identified a subpopulation of astrocytes and microglia that are Hsp25-positive in spinal cord and cortical primary mixed glial cultures. This sub-population of Hsp25-positive astrocytes in the spinal cord is 2-fold greater than that observed in cortical cultures. We also found that the expression of SOD1 ^G93A^ did not affect the regional pattern of Hsp25 expression in primary mixed glial cultures, and Hsp25 expression remained significantly higher in spinal cord cultures compared to cortical cultures. These findings were supported by findings in vivo in the SOD1 ^G93A^ mouse model of ALS, in which either a stress-induced upregulation of Hsp25 or an expansion of Hsp25-positive astrocytes was observed in the spinal cord, but not in the brain at symptomatic and late-stages of disease. The greater abundance of Hsp25-positive astrocytes in the spinal cord of SOD1 ^G93A^ mice may play a role in supporting motor neuron growth and maturation and/or may provide a cytoprotective buffer in pathological conditions. Future studies could extend on this work by investigating a range of cytoprotective glial stress responses in different CNS regions in WT and ALS mouse models. In doing so, this would further establish a link between regional variations in glial stress responses and motor neuronal vulnerability to degeneration in the spinal cords of ALS patients.

## Data Availability

The authors declare that all data underlying this research are available to the community upon request.

## Supplementary Materials

The following are available online at https://www.mdpi.com/article/10.3390/cells10051257/s1, Supplementary Figure S1: Characterisation of cortical and spinal cord mixed glial cultures. Supplementary Figure S2: Gating strategy for the analysis of Hsp25 expression of astroglia and microglia by flow cytometric analysis. Supplementary Figure S3: Heat shock stress optimisation in primary mixed glial cultures.
