# Supplementary material for: Regional Differences in Heat Shock Protein 25 Expression in Brain and Spinal Cord Astrocytes of Wild-Type and SOD1 G93A Mice
Source: Cells. 2021 May 19;10(5):1257. doi: 10.3390/cells10051257 (PMC8160835; doi:10.3390/cells10051257)
Supplement: Supplementary file 1 [file cells-10-01257-s001.zip › cells-1176158-supplementary.pdf]

Regional differences in heat shock protein 25 expression in brain and spinal cord astroglia of wildtype and $SOD1^{G93A}$ mice

Rebecca San Gil [1,2,†], Benjamin E. Clarke [3,4,†], Heath Ecroyd [1], Bernadett Kalmar [3] and Linda Greensmith [3,*]

[1] Molecular Horizons and School of Chemistry and Molecular Bioscience, Illawarra Health and Medical Research Institute, University of Wollongong, Wollongong 2519, Australia; r.sangil@uq.edu.au (R.S.G.); heathe@uow.edu.au (H.E.)
[2] Neurodegeneration Pathobiology Laboratory, Queensland Brain Institute, University of Queensland, Brisbane 4072, Australia
[3] Department of Neuromuscular Disease, UCL Queen Square Institute of Neurology, London WC1N 3BG, UK; benjamin.clarke.15@ucl.ac.uk (B.E.C.); b.kalmar@ucl.ac.uk (B.K.)
[4] The Francis Crick Institute, 1 Midland Road, London, NW1 1AT, UK

SUPPLEMENTARY INFORMATION

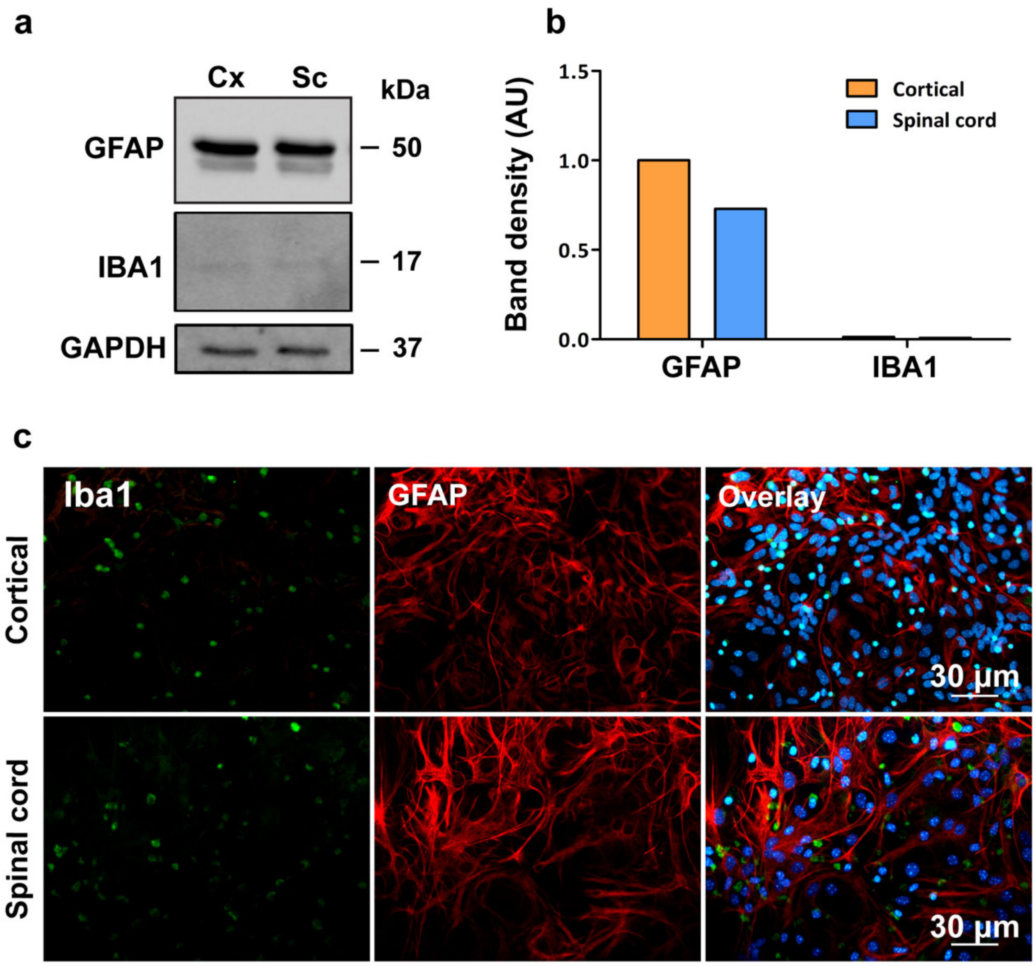

Supplementary Figure 1.. Characterisation of cortical and spinal cord mixed glial cultures.The proportion of astroglia and microglia in the mixed primary cortical and spinal cord cultures was determined after 12 days in vitro by (a-b) immunoblotting, and immunolabelling followed by (c-d) immunocytochemistry and (e-f) single cell analysis by flow cytometry. (**a**) Primary mixed glial cultures were lysed and the relative astroglial and microglial content was assessed by immunoblot analysis of GFAP and Iba1, respectively. (**b**) GFAP and Iba1 band intensity normalised to GAPDH. (**c**) Mixed primary cortical and spinal cord cultures immunolabelled with Iba1 (left), and GFAP (middle). Overlay (right) shown with DAPI nuclear stain. Scale bar = 30 µm.

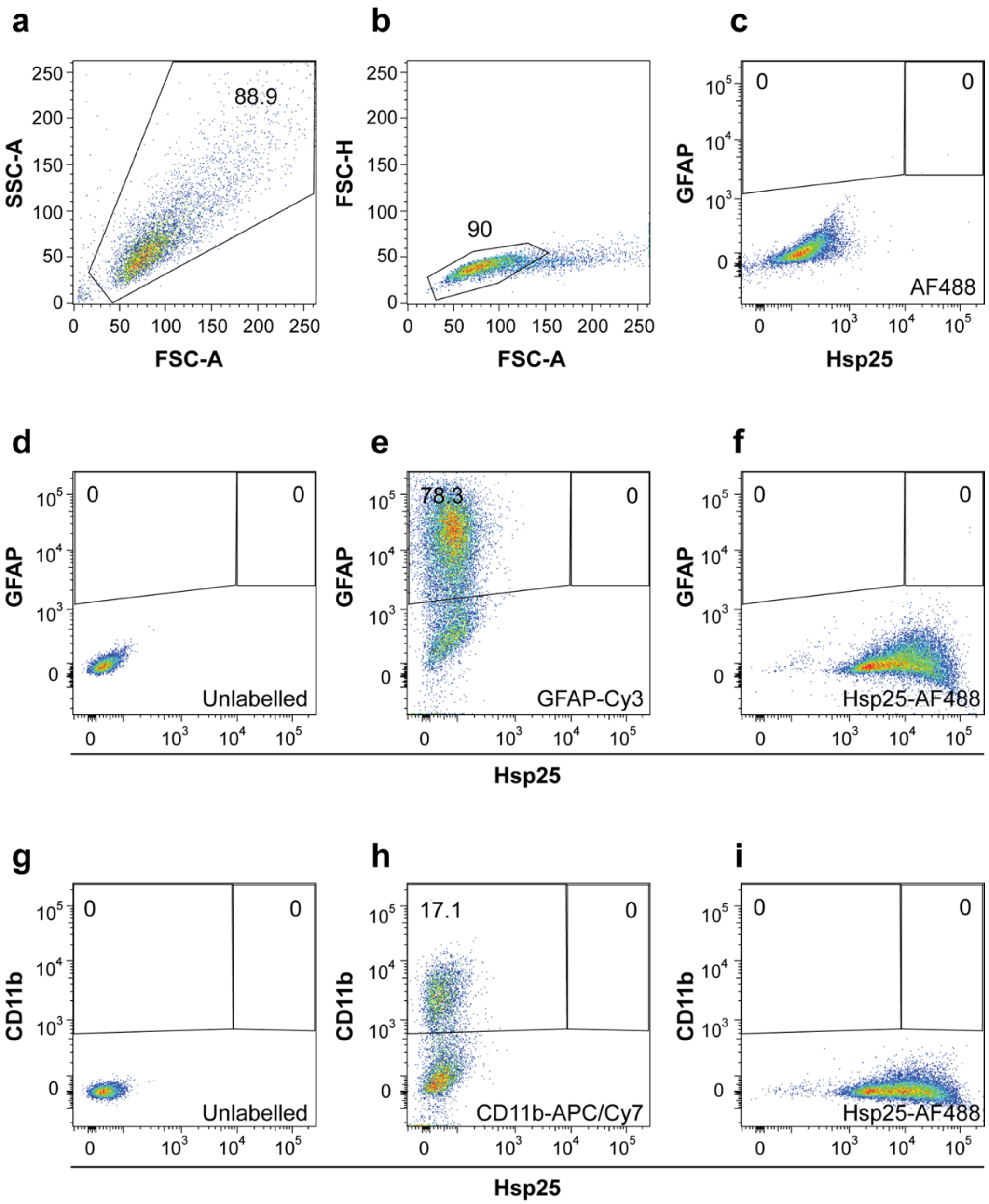

Supplementary Figure 2. Gating strategy for the analysis of Hsp25 expression of astroglia and microglia by flow cytometric analysis. (**a**) Viable cells were distinguished based on their forward scatter (area) and side scatter (area) and a polygonal gate was used to exclude cellular debris and cell clumps. (**b**) Doublets were excluded based on their forward scatter (are) and forward scatter (height). (**c**) The level of non-specific staining was determined by incubating cells with AlexaFluor 488 alone in the absence of primary antibody. Cells were also either (**d**) unlabelled or singly labelled for (**e**) GFAP-Cy3 or (**f**) Hsp25-AF488. These samples were used to set the bisected polygonal gates, which were used to determine the percent of GFAP^{+ve} and Hsp25^{+ve} events in the double labelled samples. In addition, cells were either (**g**) unlabelled or singly labelled for (**h**) CD11b-APC/Cy7 or (**i**) Hsp25-AF488. These samples were used to set the bisected polygonal gates, which were used to determine the percent of CD11b^{+ve} and Hsp25^{+ve} events in the double labelled samples.

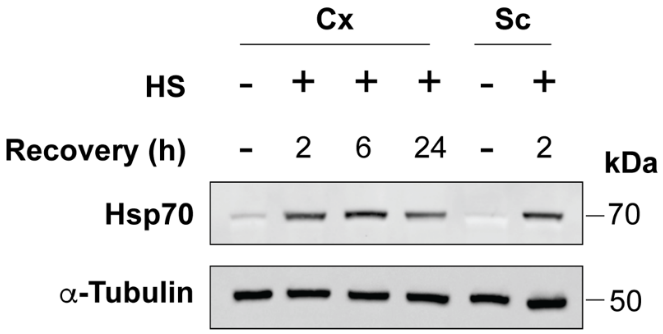

Supplementary Figure 3. Heat shock stress optimisation in primary mixed glial cultures. Primary mixed glial cultures derived from cortex (Cx) or spinal cord (Sc) were heat shocked at 42°C for 30 min and then allowed to recover at 37°C for 0, 2, 6, or 24 h. Whole cell lysates were immunoblotted for Hsp70 as a marker of heat shock response activation and a-tubulin as a loading control. .
